# Potential Role of Phytochemicals as Glucagon-like Peptide 1 Receptor (GLP-1R) Agonists in the Treatment of Diabetes Mellitus

**DOI:** 10.3390/ph17060736

**Published:** 2024-06-05

**Authors:** Julianah Ore Abiola, Ayoola Abidemi Oluyemi, Olajumoke Tolulope Idowu, Oluwatoyin Mary Oyinloye, Chukwudi Sunday Ubah, Olutunmise Victoria Owolabi, Oluwatobi T. Somade, Sunday Amos Onikanni, Basiru Olaitan Ajiboye, Foluso Oluwagbemiga Osunsanmi, Oyekanmi Nash, Olaposi Idowu Omotuyi, Babatunji Emmanuel Oyinloye

**Affiliations:** 1 Phytomedicine, Biochemical Toxicology and Biotechnology Research Laboratories, Department of Biochemistry, College of Sciences, Afe Babalola University, Ado-Ekiti 360001, Nigeria; j.orefatunmibi@gmail.com (J.O.A.);; 2 Center for Genomics Research and Innovation, National Biotechnology Development Agency, Abuja 09004, Nigeria; 3 Institute of Drug Research and Development, S.E. Bogoro Center, Afe Babalola University, Ado-Ekiti 360001, Nigeria; 4 Industrial Chemistry Unit, Department of Chemical Sciences, College of Sciences, Afe Babalola University, Ado-Ekiti 360001, Nigeria; 5 Department of Mathematics, Science and Technology Education, Faculty of Education, University of Zululand, Kwadlangezwa 3886, South Africa; 6 Department of Epidemiology and Biostatistics, College of Public Health, Temple University, Philadelphia, PA 19121, USA; 7 Medical Biochemistry Unit, College of Medicine and Health Sciences, Afe Babalola University, Ado-Ekiti 360001, Nigeria; 8 Department of Biochemistry, College of Biosciences, Federal University of Agriculture, Abeokuta 111101, Nigeria; 9 College of Medicine, Graduate Institute of Biomedical Sciences, China Medical University, Taichung 40402, Taiwan; 10 Phytomedicine and Molecular Toxicology Research Laboratory, Department of Biochemistry, Federal University Oye-Ekiti, Oye-Ekiti 371104, Nigeria; 11 Biotechnology and Structural Biology (BSB) Group, Department of Biochemistry and Microbiology, University of Zululand, Kwadlangezwa 3886, South Africa; 12 Department of Pharmacology and Toxicology, College of Pharmacy, Afe Babalola University, Ado-Ekiti 360001, Nigeria

**Keywords:** diabetes mellitus, diabetes complications, glucagon-like peptide-1 receptor agonist, phytochemicals, newer drugs

## Abstract

Currently, there is no known cure for diabetes. Different pharmaceutical therapies have been approved for the management of type 2 diabetes mellitus (T2DM), some are in clinical trials and they have been classified according to their route or mechanism of action. Insulin types, sulfonylureas, biguanides, alpha-glucosidase inhibitors, thiazolidinediones, meglitinides, sodium–glucose cotransporter type 2 inhibitors, and incretin-dependent therapies (glucagon-like peptide-1 receptor agonists: GLP-1R, and dipeptidyl peptidase 4 inhibitors: DPP-4). Although some of the currently available drugs are effective in the management of T2DM, the side effects resulting from prolonged use of these drugs remain a serious challenge. GLP-1R agonists are currently the preferred medications to include when oral metformin alone is insufficient to manage T2DM. Medicinal plants now play prominent roles in the management of various diseases globally because they are readily available and affordable as well as having limited and transient side effects. Recently, studies have reported the ability of phytochemicals to activate glucagon-like peptide-1 receptor (GLP-1R), acting as an agonist just like the GLP-1R agonist with beneficial effects in the management of T2DM. Consequently, we propose that careful exploration of phytochemicals for the development of novel therapeutic candidates as GLP-1R agonists will be a welcome breakthrough in the management of T2DM and the co-morbidities associated with T2DM.

## 1. Introduction

Diabetes mellitus (DM) is a metabolic disorder, characterized by hyperglycemia (increased blood glucose), which results from a lack of, or non-functioning, insulin thereby leaving the glucose in the bloodstream as cells are not able to take it up. The history of diabetes dates back to around 1500 BC, though it was not well understood [1], and 3500 years later, it is still a menace. It is approximated that about 537 million individuals have been diagnosed with diabetes in the year 2021 and this is predicted to increase to about 643 million people by 2030 [2]. About 75% of those living with diabetes are residents in middle- and low-income nations, while about 50% are undiagnosed. About 11.2 million Nigerians are living with DM with the highest and lowest prevalence rates seen in the South-South and North-Western zones, respectively [3].

Over the years, DM has been classified into four categories: Type 1 DM (T1DM), which usually occurs in childhood (also known as juvenile-onset), is caused by a deficiency of insulin as a result of antibodies attacking and destroying the pancreas that produces insulin. Type 2 DM (T2DM) also known as non-insulin dependent, or adult-onset has to do with insulin resistance. Here, insulin is produced, but the body cells are unable to respond to it. It is the most common of the types of DM and it takes up about 90% of all the types. Gestational diabetes is the third type, and this is seen in pregnancy as some sort of insulin resistance develops in mid to late pregnancy. It poses a threat to the fetus as excess glucose can pass through the placenta and affect the baby’s growth and development. The fourth type of DM is the group of other forms, which could result due to genetic defect, diseases of the pancreas, infections, certain surgeries, and drug use [4].

## 2. Complications Associated with Diabetes Mellitus (DM)

**Figure 1:**
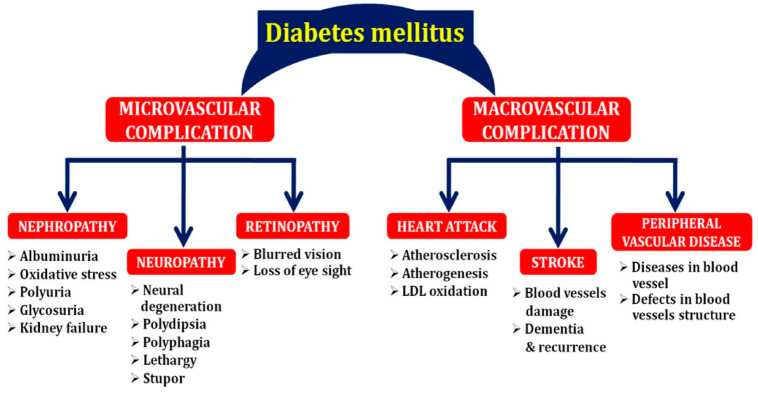
Macro- and microvascular complications in diabetes (Source: Naveen and Baskaran [6]).

Prolonged hyperglycemia is the cause of several complications such as nephropathy, neuropathy, cardiovascular disease and cancer associated with DM. The higher the amount of glycated hemoglobin (HbA1c), known as the level of glucose in the blood, the more the risk. High HbA1c can result in serious diseases affecting almost all organs of the body. There are different classifications of complications arising from DM, which include chronic and acute as well as microvascular or macrovascular complications. While chronic complications refer to diseases that build up over time as a result of prolonged hyperglycemia, acute complications are rather sudden and are usually life-threatening. Acute complications include hyperosmolar hyperglycemic state (HHS), diabetic ketoacidosis (DKA), hypers (very high blood sugar), and hypos (very low blood sugar). Chronic complications include neuropathy (nerve damage), nephropathy (kidney problems), retinopathy (eye problems), stroke/heart attack, foot problems, gum disease, and sexual problems in both men and women (Figure 1). Microvascular complications (Figure 1) include neuropathy, nephropathy, and retinopathy while macrovascular complications (Figure 1) are atherosclerosis of large vessels, which can lead to peripheral arterial disease, cardiovascular disease and stroke [5,6].

### 2.1. Nephropathy

The kidney is an important organ in the body responsible for waste removal, body fluids and blood pressure balance, and glycemic control [7]. Glucose homeostasis in the kidney occurs because it produces, releases into circulation, and uses glucose, an important energy source for the renal medulla [8]. The kidneys are made up of millions of tiny blood vessels known as glomeruli, which filter waste from the blood. Under normal conditions, the glomerulus of the kidney filters about 162 to 180 g of glucose per day with almost all the glucose being reabsorbed by sodium-glucose cotransporters (SGLTs) [7,8]. About 90% is reabsorbed by SGLT2 which is expressed in the proximal tubule while the SGLT1 transporter in the lower proximal tubule absorbs the other 10% to avoid the passing out of glucose in the urine (glycosuria) [8]. There is, however, a threshold, where the plasma glucose concentration exceeds 180 mg/dL, and the kidney is likely unable to reabsorb all the glucose, leading to the appearance of glucose in the urine.

In hyperglycemic conditions, where there is an excessive blood glucose level, the kidney reabsorbs glucose and adds to the already elevated concentrations thereby contributing to hyperglycemia. It has also been reported that glucose transporter 2 (GLUT2) and SGLT2 are upregulated in DM and glucose transport into the cells is significantly increased, suggesting that the kidney contributes to hyperglycemia through gluconeogenesis and enhanced glucose reabsorption. A study conducted by Meyer et al. [9], on the release of glucose by the liver and kidneys in diabetic animals, reported a 300% increase in renal glucose release in diabetic subjects compared to nondiabetic subjects. On the other hand, excess glucose in the blood can cause the glomeruli to be narrow and clogged, thereby blocking the free flow of blood, which can eventually damage the kidney, leading to the leakage of protein albumin out of the glomeruli into the urine. The presence of albumin in the urine is the test carried out to detect early kidney damage as there may not be any symptoms until kidney failure. Consequently, hyperglycemia affects the kidney which in turn results in more hyperglycemia until the kidney is damaged if not controlled in time.

### 2.2. Neuropathy

The inflammation and degeneration of peripheral nerves is referred to as neuropathy. Diabetic neuropathy (DN) is a collection of nerve disorders manifesting as complications of DM. Nerve damage throughout the body takes time to develop and is sometimes without symptoms in some individuals, while others may experience pain, and numbness in legs, feet, hands and arms. Nerve damage can occur in every organ system. It has been estimated that about 7 out of 10 people with DM have a type of neuropathy [10]. DN has been classified into rapidly reversible, persistent and focal/multifocal neuropathies. The rapidly reversible type is known as hyperglycemic neuropathy [11]. Another classification according to Watkins, classified neuropathies into two distinct types: those that progress as the period of diabetes increases and others that usually recover completely [12]. Autonomic and sensory neuropathies are those that mostly progress while acute, painful neuropathies, radiculopathies and mononeuropathies, although usually presented with severe symptoms, are just for a period as they tend to be reversible [13].

Of all the types of neuropathies, peripheral neuropathy (PN), also known as distal symmetric or sensorimotor neuropathy, characterized by nerve damage in the arms and legs, is the most common. PN causes muscle weakness and loss of reflexes at the ankle, leading to foot deformities, collapse of midfoot, and changes in the way a person walks. As a result of numbness in the foot, blisters, sores, and injury go unnoticed until infected, which may spread to the bone leading to eventual amputation [14].

Hyperglycemia and other metabolic factors such as impaired insulin signaling and hyperlipidemia is known to drive peripheral nerve fiber and microvessel dysfunction which leads to various downstream pathogenic pathways. It has been established that hyperglycemia contributes to oxidative stress via the overactivation of polyol, protein kinase C, and hexosamine pathways, which also occur in the nerves and microvessels. Overall, the pathophysiology of DN remains largely unknown and this is a limiting factor to the development of pathogenetic treatments [13].

### 2.3. Cardiovascular Disease

The key reason for death and disability among persons living with diabetes is cardiovascular disease (CVD) [15]. The major CVDs linked with T2DM include peripheral artery disease, coronary heart disease, stroke, heart failure and ischemic heart disease, all of which can lead to death in no less than 50% of T2DM-affected persons [16]. Hyperglycemia and resistance to insulin are usually the main characteristics of T2DM, which sometimes go with abnormal lipid metabolism. Insulin resistance is implicated in the onset and advancement of CVD and T2DM, and it is linked with an increased risk of CVD events. To reduce the occurrence of CVD, achieving a glycemic control target of less than 7% is important.

Diabetic cardiomyopathy shows changes in the heart functionally and structural changes to the left ventricle. There is an increase in the left ventricular mass compared to normal or non-diabetics which is due to the increased release of cytokines. Another proposed mechanism for the dysfunction of the cardiac system is the increased content of triglycerides in the cardiomyocytes as a result of its increased synthesis in diabetics [17].

### 2.4. Diabetes Mellitus and Cancer

It has been established already that there is a link between diabetes and cancer. DM, both types 1 and 2, increases the chance of having some kinds of cancer, and the risk is higher in women than men. There is evidence to suggest that cancer patients with diabetes have an increased risk of mortality and various infection-related morbidities [18]. Diabetes and cancer both have similar factors that exacerbate them such as aging, obesity, unhealthy diet and smoking. Several studies are trying to investigate the link between obesity and breast cancer, but with conflicting results [19].

**Figure 2:**
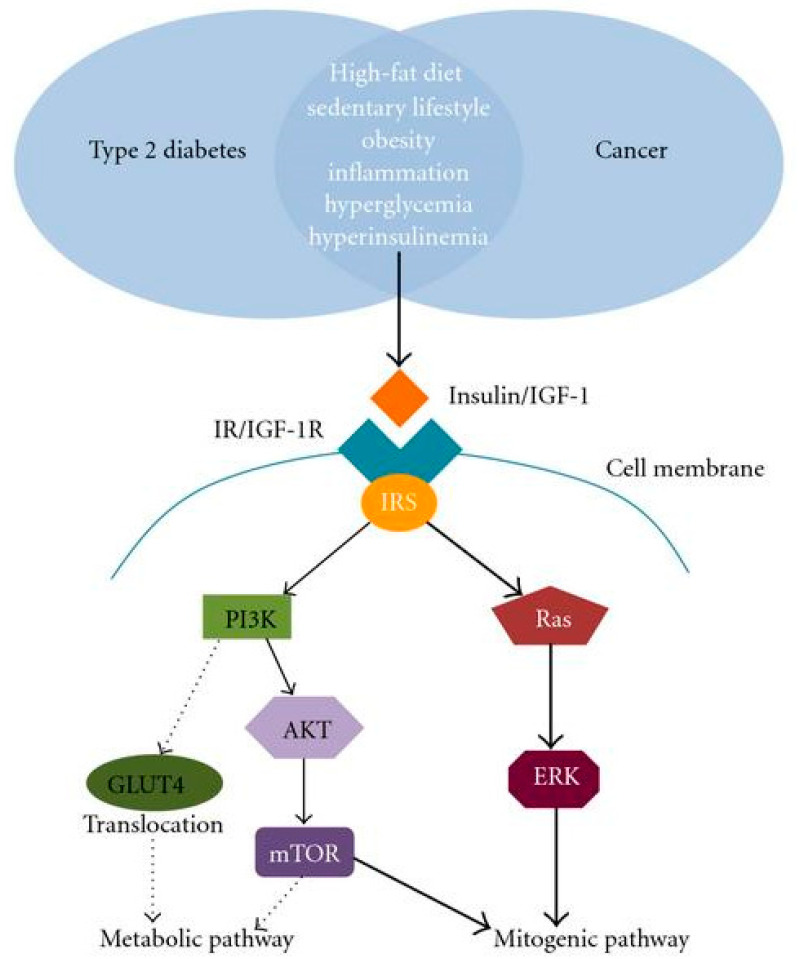
Metabolic factors linking type 2 diabetes to cancer and the activation of signaling pathways (Source: Sun and Kashyap [22]).

Nonetheless, insulin is an important hormone that activates many pathways implicated in type 2 diabetes and cancer [20]. When insulin binds to the insulin receptor (IR), the insulin receptor substrate (IRS) and the tyrosine residue of the IR are phosphorylated. IRS then phosphorylates phosphatidyl inositol-3-kinase (PI3K) which downstream activates AKT/mTOR network signaling (Figure 2). In the same vein, insulin activates insulin/insulin-like growth factor-1 (IGF-1) which leads to downstream phosphorylation cascades that activate PI3K/AKT/mTOR-network signaling and RAS/RAF/mitogen-activated protein kinase (MAPK) [21]. Studies have shown that the PI3K/AKT/mTOR-network signaling pathway is frequently activated in basal-type breast cancer [20].

Glucose enters glycolysis and Kreb’s cycle in the mitochondria to generate ATP. The process generates reactive oxygen species (ROS), which is increased in a hyperglycemic state as excess electrons convert oxygen to superoxide and then to hydrogen peroxide. High levels of ROS are a significant factor in cancer initiation and progression as DNA, proteins, and the lipid bilayer are damaged. Also, ROS has been implicated in breast cancer as pro-oncogenic pathways such as Wnt/beta-catenin, RAS and c-MYC are activated [23].

Lastly, cancer cells are highly glucose-dependent, generating their energy via aerobic glycolysis, a metabolic switch known as the “Warburg effect” as opposed to oxidative phosphorylation. This switch is needed to help the cells adapt to their hypotoxic tumor microenvironment [24]. This glucose-loving property of cancer cells would suggest that drugs that can lower blood glucose should be able to tackle cancer cells so that they are deprived and will therefore eventually die. Several studies are still on-going, while some have reported that metformin can reduce cancer incidence by 30–50% [25] and tends to impede tumor development in T2DM patients [26].

On the other hand, it is interesting to note that immunotherapy, one of many cancer treatments, may cause type 1 diabetes, although this is not common. Activated immune systems may attack beta-cells in the pancreas leading to diabetes [27].

## 3. Management of Diabetes Mellitus by GLP-1

An incretin-based remedy represents a promising and innovative method for managing diabetes for several compelling reasons. Introducing an incretin-based medication would mark a significant advancement as the first antidiabetic agent capable of stimulating insulin secretion without the drawbacks of hypoglycemia or weight gain [28]. Current studies underscore the pivotal role of GLP-1 as the primary hormone with incretin activity, which is regulated by dipeptidyl peptidase-4 (DPP4) enzymes [28]. The recognition that individuals with type 2 diabetes mellitus (T2DM) often exhibit diminished incretin responses has spurred progress in disease management. GLP-1 inducers like metformin or GLP-1 mimetics like exenatide and liraglutide have shown efficacy in improving glycated hemoglobin levels as standalone therapies or when used with other agents [28]. Notably, these agents are associated with weight reduction or are weight-neutral and carry a low risk of hypoglycemia, enhancing their clinical value. Given the short half-life of GLP-1 (2–3 min), more stable GLP-1 agonists emerge as preferred treatment options [28].

Metformin has traditionally been employed in hyperglycemia control in T2DM patients and is now recommended as a primary treatment alongside lifestyle modifications [29]. Metformin is increasingly combined with newer incretin-based medications, including GLP-1 analogues and DPP-4 inhibitors, which enhance pancreatic beta cell function [30,31]. Interestingly, some reports suggest a direct interplay between metformin and the incretin axis [32]. Studies have shown that metformin acutely increases plasma GLP-1 levels after oral glucose intake while having no significant effect on glucose-dependent insulinotropic polypeptide (GIP) or peptide YY levels [33,34,35,36]. Metformin’s impact on the gut endocrine system appears to be L-cell specific, primarily affecting GLP-1 secretion [37,38]. Additionally, metformin may inhibit the apical sodium-dependent bile acid transporter, potentially stimulating GLP-1 secretion through TGR5 activation [37,38]. Another proposed mechanism involves metformin inhibiting DPP-4 activity, leading to increased plasma GLP-1 levels [39,40].

Exenatide, a synthetic analogue of exendin-4 derived from the Gila monster, shares a similar embryonic origin with human GLP-1 but is immune to DPP-4 degradation [41,42]. FDA and EMEA approvals for exenatide include use as an add-on therapy to metformin, sulphonylureas, or thiazolidinediones for T2DM treatment [43]. Reported side effects of exenatide include nausea, vomiting, and rare cases of pancreatitis [44].

Liraglutide, another synthetic analogue of human GLP-1, exhibits resistance to DPP-4 degradation and demonstrates safety profiles unaffected by renal or hepatic impairment [33,34]. Despite the benefits of GLP-1 agonists in diabetes management, concerns about severe side effects like pancreatitis, and renal and hepatic impairments have emerged.

## 4. Intracellular Signaling Pathway of GLP-1R

GLP-1 functions within the pancreas to lower blood glucose levels through various mechanisms. It works by enhancing insulin synthesis and release, promoting neogenesis and proliferation, and reducing apoptosis of β cells [45]. The process of GLP-1-induced insulin secretion from pancreatic β cells was extensively researched as the principal intracellular signaling pathway mediated by GLP-1r [45].

**Figure 3:**
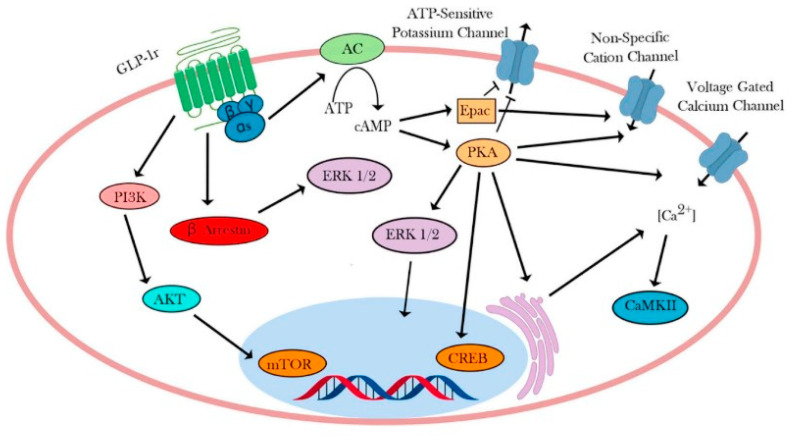
GLP-1R-controlled signaling (Source: Smith et al. [45]).

In this pathway, GLP-1r acts via Gαs to activate adenylate cyclase, leading to increased levels of cyclic AMP (cAMP) [46]. Elevated cAMP levels facilitate protein kinase A (PKA)-dependent intracellular signaling and exchange proteins directly activated by cAMP (EPAC) processes. Activation of these pathways enables GLP-1 to trigger various cellular mechanisms that culminate in insulin release and genetic modifications [47,48,49,50,51]. GLP-1 is demonstrated to operate through PKA and EPAC (cAMP-dependent pathways) to inhibit ATP-regulated potassium channels [52,53,54,55], boost the activity of L-type voltage-gated calcium channels (VGCCs) [56,57], and induce the opening of non-specific cation channels (refer to Figure 3) [58,59]. These combined actions promote calcium influx, consequently enhancing calcium-induced insulin secretion. Notably, blocking ATP-regulated potassium channels leads to heightened glucose-induced membrane depolarization, thereby increasing cellular sensitivity to glucose [60]. There is limited evidence suggesting that analogous mechanisms may occur in GLP-1r-expressing neurons in the hippocampus and hypothalamus [61,62,63].

## 5. Agonists of GLP-1R

Glucagon-like peptide-1 (GLP-1) agonists, also known as GLP-1 receptor agonists or incretin mimetics, are medicines used in managing type 2 diabetes mellitus and sometimes obesity. Examples include exenatide, lixisenatide, liraglutide, albiglutide, dulaglutide, and semaglutide [64]. Metformin remains the preferred initial treatment for type 2 diabetes according to the American Diabetes Association. However, adding a GLP-1 analog is recommended for patients who cannot tolerate or have contraindications to metformin, those whose hemoglobin A1c is more than 1.5% above target, or individuals not achieving their A1c goal within three months, especially those with atherosclerosis, heart failure, or chronic kidney disease [65,66,67]. Additionally, semaglutide and high-dose liraglutide are FDA-approved for obesity treatment and can be prescribed to obese patients with comorbidities. Research suggests that GLP-1 analogs may benefit type 1 diabetes patients by improving hemoglobin A1c levels and promoting weight loss. However, high costs and tolerability issues remain barriers to broader use [68,69,70].

Structurally, GLP-1 agonists can be categorized into two groups: those with a human GLP-1 backbone (including dulaglutide, albiglutide, liraglutide, and semaglutide) and those with an exendin-4 backbone (such as exenatide in its various formulations and lixisenatide) [64].

Tirzepatide is a glucagon-like peptide-1 (GLP-1) and glucose-dependent insulinotropic polypeptide (GIP) receptor dual agonist. Safety concerns have led to the discontinuation of research on another agent, taspoglutide, during phase III trials [71].

### 5.1. GLP-R1 Agonists and Their Importance in Managing Diabetes

#### 5.1.1. Brain and Heart Protection

GLP-1 receptor agonists intervene in several molecular and cellular stages of atherogenesis. GLP-1 plays pivotal roles in diminishing reactive oxygen species production, platelet activation, macrophage and monocyte activation, and subsequent accumulation in vascular walls, as well as inhibiting endothelin production, leading to vasodilation. GLP-1 receptor agonists amplify these beneficial actions [72,73]. Additionally, these medications stabilize endothelial cells, reducing plaque hemorrhage and rupture ultimately decelerating atherosclerosis progression [74,75].

#### 5.1.2. Kidney Protection

The precise mechanisms responsible for the GLP-1 receptor agonists’ renal protective effects are not entirely clear. However, these drugs lower hemoglobin A1c, weight, and blood pressure, thereby modifying the conventional risk factors for chronic kidney disease and the progression of diabetic nephropathy [76]. Furthermore, GLP-1 receptors are present in the cells of renal proximal convoluted tubules and preglomerular vascular smooth muscle, and their direct stimulation inhibits the sodium–hydrogen exchanger 3 at the brush border of proximal convoluted tubular cells. This results in increased natriuresis and consequently reduced blood pressure.

#### 5.1.3. Effect on Weight

In rat studies, GLP-1 receptor agonists stimulate hypothalamic GLP-1 receptors thereby preventing meal initiation and inducing meal termination [77]. Human studies also demonstrated that patients receiving GLP-1 receptor agonists experienced reduced energy intake, suppressed appetite, and diminished food cravings. These patients exhibited altered taste preferences, a decreased inclination towards fatty and energy-dense foods, and reduced pleasure in eating [78]. These hypothalamic effects may differ among patients treated with GLP-1 receptor agonists.

## 6. Medicinal Plants

Medicinal plants, also referred to as phytomedicinals, are plants which can be administered as a part or whole, in the form of tea, extract or tinctures, to treat illness. The use of plants as medicine has a long history; for instance, willow (*Salix* sp.) has been used for medical purposes for 6000 years [79], while the synthetic drug aspirin was created from salicylic acid extracted from willow bark in 1987 [80]. Primary and secondary metabolites are two categories of chemical molecules produced by plants and generally known as phytochemicals. Secondary metabolites have a variety of roles in many aspects of plant life, including competition, protection from disease and damage, and species interaction. Primary metabolites aid in plant development and metabolism. Carbohydrates, proteins, lipids, amino acids, purines, and pyrimidines of nucleic acids are examples of primary metabolites. According to Rabikadeh et al. [81], secondary metabolites are substances produced by the cell from the primary metabolic pathways and have been reported to have antifungal, antiviral, and antibiotic properties.

Phytochemicals not only have therapeutic effects but also have additional properties such as promoting health, preventing disease, and supplying nutrients to the body which allow them to be considered as functional foods or nutraceuticals [82]. Foods capable of performing required functions are natural or processed food products, which contain essential nutrients that help the body to prevent, and/or manage the treatment of diseases [83]. Studies have shown that a group of phytochemicals such as organosulfur compounds, including allium compounds and glucosinolates, carotenoids, phytosterols, phytostanols and phenolic compounds (phenolic acids, flavonoids, phytoestrogens) prevent certain chronic diseases, e.g., CVDs, cancer, and diabetes, when consumed [84]. For instance, flavonoids, a subclass of polyphenols, provide health benefits by scavenging free radicals and regenerating other dietary antioxidants and chelate pro-oxidant metals [85]. They are known to prevent CVDs, diabetes, and osteoporosis and treat wound healing [86]. Also, a phytochemical in cruciferous vegetables known as glucosinolates was found to protect against cancer of the stomach, rectum, and colon [87]. The various classes of phytochemicals in medicinal plants have made possible their diverse functions, in addition to therapeutics and functional foods, such as cosmetics, fragrances, supplements, etc.

### 6.1. Class of Plant Phytochemicals

**Figure 4:**
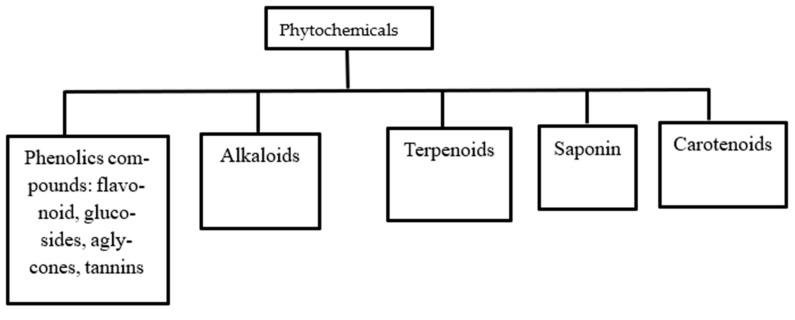
Classes of phytochemicals.

There is no specific classification of the secondary metabolites of plants but they have been grouped according to their structure as shown in Figure 4. The groups include phenolic compounds, alkaloids, terpenoids, saponins, and carotenoids.

#### 6.1.1. Phenolic Compounds

They are the most widely distributed compounds in plants with an -OH group bonded to an aromatic hydrocarbon group [83]. The majority of plant phenolic chemicals are flavonoids; other types include glucosides and aglycones. Tannins are phenolic polymers. Numerous pharmacological effects, including antibacterial, anti-inflammatory, anti-tumor, and cytotoxic properties, have been linked to flavonoids. Since they scavenge reactive oxygen species and free radicals, they are well-recognized and referred to as antioxidants [88].

#### 6.1.2. Alkaloids

The name is from “alkaline” as they are basic in character. These are naturally occurring substances, having heterocyclic nitrogen atoms and an unpleasant taste. One such is quinine, which has a bitter taste. They are categorized by the nature of the heterocyclic ring present in their structure such as pyrrolidine, pyridine–piperidine, and isoquinoline alkaloids. They are used in neuro-pharmaceuticals in anti-cancer, sedative, anti-microbial roles, and as insecticides [88].

#### 6.1.3. Terpenoids

Using isoprene (CH_2_=C(CH_3_)-CH=CH_2_) as their building block, they are components of essential oils that are used in food and cosmetics as flavors and perfumes. The majority of them contain basic carbon skeletons and multicyclic structures with various functional groups. They are divided into six groups: sesquiterpenes, diterpenes, triterpenes, tetraterpenoids, hemiterpenoids, and monoterpenoids. They have hepaticidal, anti-microbial, detoxifying, strengthening, anti-rheumatic, and anti-malarial properties [88,89].

#### 6.1.4. Saponin

The term “saponin” comes from the stable foam they produce in aqueous solutions, similar to soap. Triterpenoids, steroid alkaloids, and glycosylated steroids are examples of saponin. Most saponins are considered as a component of a plant’s defensive mechanism since they are known to shield plants against insect damage [90]. However, they have been researched and found to be hypocholesterolemic, anti-carcinogenic, hypoglycemic, antifungal, and antiviral [88].

#### 6.1.5. Carotenoids

They are fat-soluble pigments which could be yellow, orange or red. They are divided into two major classes: those that have oxygen-free hydrocarbons, like lycopene, beta- and alpha-carotene, and those that have oxygenated hydrocarbons, e.g., xanthophylls. They are known to prevent eye diseases and protect against carcinogens in the breast, liver, colon, brain, cervix, and prostate [83].

The pathogenesis of several human diseases such as CVD, some cancers, and aging are linked to excessive production of oxidants in the body leading to oxidative damage. Antioxidant phytochemicals have been studied and are known to prevent and treat diseases [91]. For instance, the phytochemical class known as polyphenol chelates to pro-oxidant metals and produces and protects dietary antioxidants like vitamin E, and eliminates free radicals. Turmeric’s hydrophobic polyphenol, curcumin, protects the skin from damage by scavenging free radicals and lowering inflammation by blocking NF-ҡB [92]. Chronic inflammation is another component that contributes to the development of chronic diseases including CVD, cancer, and DM. Studies have demonstrated the anti-inflammatory and anti-neoplastic cell growth effects of curcumin through downregulating survivin and IGF-1 (insulin growth factor) expression, upregulating p53 expression, and lowering tumor necrosis factor-α (TNF-α) levels, which triggers apoptotic signals [93,94].

As previously noted, there is a connection between inflammation, obesity, and diabetes. According to research, curcumin reduces leukocyte adherence to the endothelium, which reduces vascular inflammation in rats with diabetes. It also reduces reactive oxygen species (ROS) by downregulating increased levels of malondialdehyde (MDA) [95]. Furthermore, other researchers found that curcumin decreased NF-ҡB activation and IL-1β production in the retina of diabetic rats, indicating that curcumin may have therapeutic benefits for diabetic retinopathy [96]. Baicalein, a flavone derived from *Scutellaria baicalensis*, was found to have an impact on renal inflammatory processes by lowering the expression of TGF-β, iNOS, and NF-ҡB in the kidney, suggesting its effect in diabetic nephropathy [97].

### 6.2. Possible GLP-1-Inducing Mechanism by Phytochemicals

**Figure 5:**
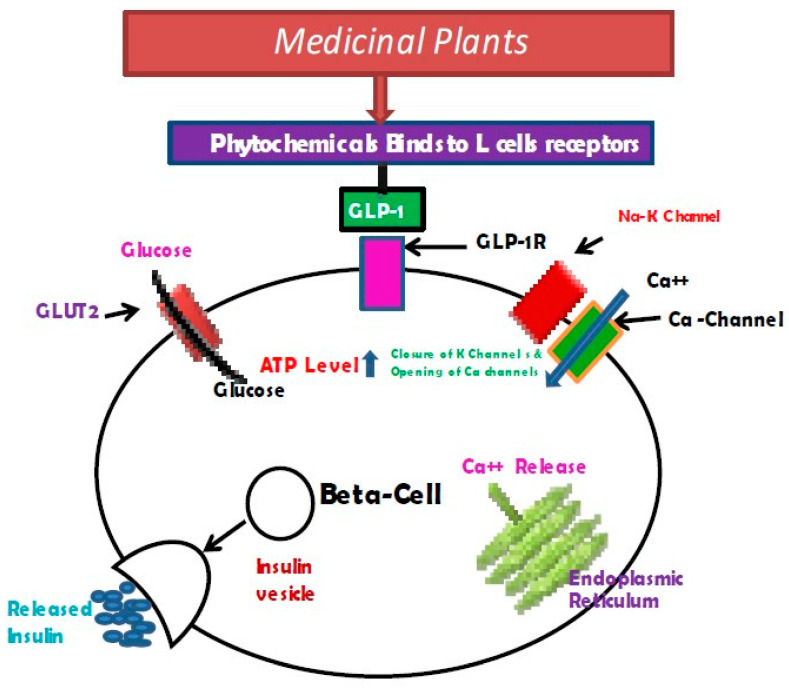
Mechanism of induction of GLP-1 by phytochemicals (Source: Singh et al. [28]).

Phytochemicals have been observed to potentially stimulate the GLP-1 receptor on enteroendocrine cells within the gut, initiating a cascade of signal transduction events involving key proteins such as G protein-gustducin, phospholipase C beta 2 (PLCβ2), inositol 1,4,5-trisphosphate receptor type 3 (IP3R3), and transient receptor potential (TRP) channels [28]. These processes ultimately lead to the depolarization of the enteroendocrine cell membrane by increasing intracellular Ca2+ levels, resulting in the release of GLP-1 [28]. Figure 5 illustrates a schematic representation of the GLP-1-mediated insulin secretion from beta cells.

### 6.3. Role of Medicinal Plants in Enhancing GLP-1 Level

The plant kingdom presents a significant flair for the discovery of novel medicines to treat various diseases, which include diabetes mellitus (DM). According to a literature search and survey, approximately 400 plants and 700 plant-based recipes have been documented worldwide for managing DM [98]. The mechanisms of action of medicinal plants in diabetes management include the regeneration of β cells and the improvement of insulin secretion from the pancreas, increased glucose uptake by muscles and adipose tissue, decreased gluconeogenesis, and the inhibition of intestinal α-glucosidase [99]. Recent studies have identified GLP-1 modulatory activity in medicinal plants [100,101,102], which holds promise for diabetes management. Table 1 provides an overview of medicinal plants with GLP-1-inducing activity.

### 6.4. Phytochemicals and GLP-1

Extensive studies have been carried out on some phytochemicals in different diabetic models to understand their mechanism of action (Table 2). One of these is the study of Cicero and Tartagni [114] on the antidiabetic effect of berberine, a phytochemical from *Berberis vulgaris* root/rhizome. They reported that at 500 mg/kg body weight in rat, berberine increased insulin secretion, induced glycolysis while also increasing the levels of GLP-1 and glucose transporter-4 (GLUT-4).

Additionally, studies reported that *geniposide* from *Gardenia jasminoides* fruit in INS-1 cells, which secrete insulin, improves glucose-stimulated insulin secretion via the activation of the glucagon-like peptide 1 receptor (GLP-1R) and prevents oxidative stress-induced neuron death [103,127]. In vitro, GLP-1 binding to its receptor was enhanced by tarralin, another phytochemical found in Artemisia dracunculus leaves, when given to KK-A (gamma) mice at a dose of 500 mg/kg [105].

According to Singh et al. [28], one possible mechanism for GLP-1 induction by phytochemicals involves activating GLP-1R on the gut’s enteroendocrine cells, which activates many signal transducers, including inositol 1,4,5-triphosphate receptor type 3 (IP3R3), G protein α-gustducin, phospholipase C beta 2 (PLCβ2), and transient receptor potential (TRP) channels. These actions depolarize the membrane of the enteroendocrine cell membrane by releasing GLP-1 and increasing intracellular Ca^2+^ concentration.

### 6.5. Flavonoids as GLP-1R Agonists

Several flavonoids have been shown to enhance GLP-1 secretion in intestinal cell models and tissues, leading to increased plasma GLP-1 concentrations in animal studies. Most animal experiments indicate improved glucose tolerance alongside elevated plasma GLP-1 levels. Studies using enteroendocrine cell models such as murine GLUTag and human NCI-H716 cells, curcumin, delphinidin 3-rutinoside, ginsenoside metabolite Rg3, hispidulin, and isoquercitrin demonstrated stimulatory effects on GLP-1 secretion (Table 3) [122,123,124,125,126,127,128,129,130]. Although less recognized, human Caco2 cells were also used to illustrate EGCG-induced GLP-1 secretion (reference [131]). This study also showcased the GLP-1-releasing activity of EGCG in a mouse intestinal tissue segment model.

In animal experiments, single doses of curcumin [122] and the ginsenoside metabolite Rg3 [129] have been reported to induce GLP-1 secretion. Additionally, chronic administration (either orally or intraperitoneally) of apigenin [133], genistein in combination with metformin [136], hispidulin [126], isoquercitrin [130], luteolin [139], myricetin [138], grape seed proanthocyanidins [135], procyanidin [137], and resveratrol [134] has been reported to increase plasma GLP-1 levels. Some compounds, such as isoquercitrin and myricetin, are reported to inhibit the DPP-4 enzyme, which may contribute to their ability to promote GLP-1 levels [130,138]. Flavonoids exhibit diverse protective effects against obesity, diabetes, and other metabolic disorders by targeting various organs, tissues, and cells, as discussed previously. Therefore, the promotion of GLP-1 by flavonoids may partly underlie their health-promoting effects.

### 6.6. Alkaloids as GLP-1R Agonists

#### Berberine

Berberine, an isoquinoline alkaloid, found in several plants like goldenseal and barberry, demonstrated GLP-1R agonist activity in studies, leading to enhanced glucose metabolism and insulin sensitivity in animal and cell culture models [140]. Berberine induces GLP-1 secretion in the intestine by modulating the gut microbiota, potentially alleviating diabetes symptoms. Additionally, berberine decreases mitochondrial stress and cytochrome c relocation out of the mitochondria. The production of short-chain fatty acids (SCFAs) induced by berberine also contributes to GLP-1 secretion from intestinal L-cells [140].

## 7. Survival Proteins of β-Cells Revealed by GLP-1RAs

Extensive research has focused on elucidating how GLP-1 receptor agonists (GLP-1RAs) counteract the detrimental effects of ER stress [141], oxidative stress [142], and autophagy [143]. These investigations have identified critical targets necessary for β-cell protection. The proteins identified in Table 4 meet specific criteria for β-cell protection elicited by GLP-1RAs: they are activated and/or induced by GLP-1RAs in β-cells, and their inhibition and/or suppression diminish the protective effects of GLP-1RAs against cell death induced by pro-apoptotic stressors. These proteins form part of the GLP-1RA signaling cascade and are interconnected, as illustrated by the IB1/JIP1/JNK3 pathway [144]. Consequently, targeting these proteins with GLP-1RAs represents a promising therapeutic approach for enhancing β-cell mass in type 2 diabetes.

## 8. Traditional Medicine and GLP-1R Agonists

Traditional medicine or the use of herbs/ medicinal plants in the treatment of diseases is an ancient practice. To date, two ancient traditional medicinal systems stand out; Ayurvedic medicine which is a highly recommended form of medicine in India [151], and Traditional Chinese Medicine (TCM) [152]. Recently, Africa has stepped up the game in coming up with evidence-based traditional medicine; however, there are still limitations.

### 8.1. Ayurvedic Antidiabetic Plants with GLP-1R Agonism

In Ayurvedic medicine, *Madhumeha*, one of the four types of *Prameha* is similar clinically to present-day diabetes mellitus. From the name, “*madhu*” means sweetness and “*meha*” means urination [151]. Many local Indian medicinal plants have been said to be a success in the management of diabetes such as *Fructus corni* (*Cornus officinalis*), *Fructus schisandrae*, *Rhizoma alismatis*, *Rhizoma dioscoreae*, *Gymnema sylvestre* (gudmar)*, Momordica charantia* (karela)*, Pterocarpus marsupium* (Beejsar/Vijaysar), *Rubia cordifolia*, *Syzygium cumini* (jamun)*, Brassica juncea, Curcuma longa, Ficus glomerate, Acacia arabica* (babul), *Aegle marmelos* (bael), *Agrimonia eupatoria* (church steeples), *Allium cepa* (onion), *Allium sativum* (garlic), *Aloe vera* (Ghrit kumari), *Azadirachta indica* (neem), *Beta vulgarus* (beetroot), *Benincasa hispida* (ash gourd), *Caesalpinia bonducella* (fever nut), *Citrullus colocynthis* (bitter apple), *Coccinia indica* (ivy gourd), *Eucalyptus globulus* (eucalyptus), *Ficus benghalensis* (banyan tree), *Hibiscus rosa chinensis* (Gurhal/Jaswandi), *Ipomoea batatas* (sweet potato), *Jatropha curcas* (purging nut), *Mangifera indica* (mango), *Morus alba* (mulberry), *Mucuna pruriens* (kiwach), *Ocimum sanctum* (tulsi), *Punica granatum* (anar), *Tinospora cordifolia* (giloy), and *Trigonella foenum-graecum* (methi) [151].

Phytochemicals from some of these plants have been identified through studies to stimulate GLP-1 secretion such as *Momordica charantia* [110] and *Curcuma longa* [132] while some have a GLP-1 receptor agonism property. *Cornus officinalis* is used in Ayurvedic and TCM in the management of vascular complications of DM and many other diseases. Xu et al. reported that a secoiridoid glycoside named Morroniside from the plant activates GLP-1 receptor [153] and Park et al. reported its effect on diabetes-induced alterations [154].

### 8.2. Traditional Chinese Medicine Antidiabetics and GLP-1 Receptor Agonist

According to TCM general principles, diabetes impairs “qi (energy/life-force)” causing reduced circulatory system function. The spleen, in TCM, is the source of vital energy and blood and it controls its circulation; weakness to the spleen’s qi spirals down to damage kidney endothelial cells and eventually produces renal fibrosis. Therefore, in treating diabetes, qi and circulatory function must be improved; hence, most prescribed formulations are spleen strengtheners (156). One common formula used in TCM in treating diabetes is Rehmannia Six Formula (RF) which contains six herbs; *Rehmannia glutinosa*, *Fructus corni*, *Dioscorea, Poria cocos*, *Alisma* and *Paeonia suffruticosa.* Of all the herbs, *Fructus corni* is the only plant with a compound able to activate the GLP-1 receptor. Also, Tang et al. reported that Wenyujinoside and 28-deglucosylchikusetsusaponin IV could activate GLP-1 receptor for DM treatment [155]. Wenyujinoside is from *Curcuma wenyujin* [156] while 28-deglucosylchikusetsusaponin IV is from *Panax japonicus* [157].

### 8.3. African Traditional Treatments of Diabetes

Mohammed and Tajuddeen reported that about 82 compounds from 24 different plants across Africa were studied over 5 years (2015–2020). Some of the compounds include quercetin, epicatechin, protocatechuic acid, kolaviron, oleanolic acid, ursolic acid and lupeol while some of the plants include *Aframomum melegueta* (Nigeria), *Antidesma madagascariense* (Madagascar), *Leonotis ocymifolia* (Namibia), *Myrianthus arboreus* (Ghana), and *Ziziphus mucronate* (South Africa) [158]. Preparation of the plants usually involves boiling fresh leaves or stems in the form of tea, extracting the juice from the leaves to take orally, dried fruit or seed ground into powder to take with pap [158]. Various studies suggested the mechanism of action for these compounds in the management of DM; however, none was reported to be a GLP-1 receptor agonist. Continuing research on local plants with antidiabetic potential, especially to elucidate their mechanism of action, and then advancing from preclinical results to clinical trials with financial support from the relevant industries and the Government would go a long way to help African traditional medicine.

## 9. Conclusions

Medicinal plants are being used worldwide, especially by those who consider them the most readily available and the cheapest. However, they are not yet accepted by all, based on several schools of thought, some of which may be true or false. T2DM remains a multifactorial disease which will need a combination of multi-targeted molecules as treatment. The use of medicinal plants would be a better approach in solving this dilemma, especially with further intensive research and policies being put in place to make it more widely accepted and not just seen as an alternative.

Although these phytochemicals have shown potential effects on the glucose metabolism and insulin sensitivity in experimental models, their specific mechanisms of action and interactions with GLP-1R signaling may differ and warrant additional investigation. Introducing a variety of phytochemical-rich foods into one’s diet could provide advantages for metabolic health; however, further studies are necessary to comprehensively grasp their therapeutic implications for conditions such as diabetes and obesity.

## Figures and Tables

**Table 1:** Some GLP-1-inducing medicinal plants.

Common Name	Used Part	Phytochemicals	Mechanism	References
Gardenia	Fruit	Geniposide	Geniposide has been shown to protect against neuronal apoptosis induced by oxidative stress and enhance glucose-stimulated insulin secretion via activation of the glucagon-like peptide 1 receptor (GLP-1R) in INS-1 cells.	Liu et al. [103]
Cinnamon tree	Bark	Cinnamon	Consuming 3 g of cinnamon resulted in decreased postprandial serum insulin levels and increased concentrations of GLP-1, with no significant impact on blood glucose levels.	Hlebowicz et al. [104]
Mate tea	Leaves	Mate-saponin 2, matesaponin, 3,5-O-dicaffeoyl-D-quinic acid	The acute administration of key components of mate led to notable GLP-1 increased levels. Specifically, compounds such as 3,5-O-dicaffeoyl-D-quinic acid and matesaponin 2, along with alpha-linolenic acid, demonstrated significant enhancements in GLP-1 levels.	Hussein et al. [100]
Little dragon	Leaves	Tarralin	The extract demonstrated enhancement in the binding of glucagon-like peptide (GLP-1) to its receptor in in vitro studies.	Ribnicky et al. [105]
Soybean	Roots	Glyceollins	Glyceollins were found to enhance GLP-1 secretion, thereby amplifying insulinotropic effects in enteroendocrine cells.	Park et al. [106]
Agave	Roots	Agave fructans	Agave fructans has been shown to increase GLP-1 levels and enhance the concentration of its precursors.	Urias-Silvas et al. [107]
Yacon	Roots	Fructooligosaccharides	Diabetic rats treated with a yacon flour-supplemented diet exhibited a significant increase in glucagon-like peptide-1 (GLP-1) content compared to diabetic control rats.	Habib et al. [108]
Pygeum	Bark		This plant is concluded to enhance insulin secretion by reducing DPP-4 activity, thereby prolonging the half-life of GLP-1.	Suleiman [109]
Bitter melon	Fruit	Karavilagenine E	Mice that received a single oral dose of WES for 30 min exhibited higher serum levels of GLP-1 and insulin, along with lower glucose levels. This suggests that WES stimulates GLP-1 secretion in vivo.	Huang et al. [110]
Wheat	Fibers		Consuming more wheat fiber over an extended period leads to an eventual increase in the production of short-chain fatty acids (SCFA) and the secretion of glucagon-like peptide-1 (GLP-1).	Freeland et al. [111]
Mango	Leaves		*Mangifera indica* inhibits DPP-4 and enhances GLP-1 levels in individuals with type 2 diabetes mellitus (T2DM).	Yogisha and Raveesha [112]
Korean pine	Seed	Triglyceride and free fatty acids	GLP-1 levels were observed to be higher 60 min after the introduction of pine nuts.	Pasman et al. [113]
Barberry	Roots, rhizomes	Berberine	The antidiabetic effect of berberine is attributed to its ability to increase insulin secretion, promote glycolysis, and elevate levels of glucose transporter-4 (GLUT-4) and glucagon-like peptide-1 (GLP-1).	Cicero and Tartagni [114]

**Table 2:** Phytochemicals from medicinal plants that serve as GLP-1R agonist.

	Plants	Activities	Methods	Name of Compound	References
1.	*Anoectochilus roxburghii*	In vivo	Restoration of damaged β cells in pancreas	Kinsenoside	Zhang et al. [115]
2.	*Bacopa monnieri* (L.) *Wettst.*	In vivo	Consumption of peripheral glucose and protection against oxidative damage	Bacosine	Ghosh et al. [116]
3.	*Berberis aristata*	In vivo	Regulates glucose homeostasis by reducing gluconeogenesis and oxidative stress	Berberine	Potdar et al. [117]
4.	*Berberis vulgaris*	In vivo	Increases insulin secretion and stimulates glycolysis	Berberine	Cicero and Tartagni [114]
5.	*Gardenia jasminoides J. Ellis*		Phosphorylation of Akt and FOXO1 in INS-1 cells	Geniposide	Liu et al. [103]
6.	*Artemisia dracunculus* L.	In vitro	Lessens the secretion of glucagon	Tarralin	Ribnicky et al. [105]
7.	*Aegle* *marmelos* *Correa*	In vivo	Stimulates insulin secretion from β cells	Coumarins	Ruhil et al. [118]
8.	*Zingiber* *officinale* *Roscoe*	In vivo	Enhanced ability to withstand glucose and support the release of insulin induced by glucose	6-gingerol	Samad et al., [119]
9.	*Bumelia* *sartorum Mart.*	In vivo	Increase in glucose uptake and glycogen synthesis. Increase in the amount of insulin secreted by pancreatic beta-cells	Bassic acid	Naik et al. [120]
10	*Nigella sativa* L.	In vivo	Potential stimulation in pancreatic β-cells causing insulin secretion, reduced hepatic gluconeogenesis	Thymoquinone, Dithymoquinone	Benhaddou-Andaloussi et al. [121]
11	*Agave tequilana F.A.C. Weber*	In vivo	Improved lipid glucose metabolism by inducing proglucagon	Fructans	Urias-Silvas et al. [107]
12	*Panax ginseng C.A. Mey*	In vitro and in vivo	Upregulation of proglucagon gene expression and glucose induced GLP-1	Saponins and Ginsenoside	Liu et al. [103]
13	*Cinnamomum verum J. Presl.*		Increased blood GLP-1 concentration	Cinnamon	Hlebowicz et al. [104]
14	*Curcuma longa* L.	In vitro	Increased GLP-1 secretion	Curcumin	Takikawa et al. [122]
15	*Cynanchum marnierianum Rauh*	In vitro	Stimulates GLP-1 secretion	Pregnane glycoside	Tsoukalas et al. [123]
16	*Glycine max* (L.)	In vitro	Dose-dependent increase in GLP-1 secretion	Glyceollins	Park et al. [106]
17	*Hibiscus sabdariffa* L.		Elevation of GLP-1 in ileum and pancreas	Delphinidin	Kartinah et al. [124]
18	*Hoodia gordonii (Masson)*	In vivo	Induces the release of GLP-1 via GPR119	Gordonoside F	Zhang et al. [91]
19	*Momordica charantia*	In vivo	Induces plasma GLP-1 and stimulates insulin release	Cucurbiracin	Dans et al. [125]
20	*Rheum palmatum* L.	In vivo and in vitro	Increased plasma GLP-1 secretion	Emodin	Wang et al. [126]

**Table 3:** Flavonoids possessing GLP-1-elevating effect.

Compound	Models	Effect	Reference
Curcumin	Rats	Elevated GLP-1 in plasma, elevated tolerance to glucose	Kato et al. [132]
Epigallocatechin-3-gallate	Murine ileal tissue and caco-2 cells	Elevated secretion of GLP-1	Song et al. [131]
Delphinidin 3-rutinoside	GLUTag cells	Elevated secretion of GLP-1	Kato et al. [128]
Apigenin	High-fructose and -fat diet rats	Elevated GLP-1 in plasma	Kalivarathan et al. [133]
Curcumin	GLUTag cells	Elevated secretion of GLP-1	Takikawa et al. [122]
Resveratrol	High-fat diet mice	Elevated GLP-1 in plasma, elevated tolerance to glucose	Dao et al. [134]
Hispidulin	STZ-treated mice	Elevated GLP-1 in plasma, elevated tolerance to glucose	Wang et al. [126]
Gallic acid	Ileal segment of rat	Elevated secretion of GLP-1	Casanova-Marti et al. [135]
Genistein-metformin	Alloxan-induced diabetic rats	Elevated intestinal and serum GLP-1	Rehman et al. [136]
Isoquercitrin	High fat diet; streptozotocin-administered rats	Elevated plasma glucose and GLP-1; reduced plasma DPP-4	Zhang et al. [130]
Procyanidin	Cafeteria-diet rats	Elevated intestinal GLP-1	Gonzalez-Abuin et al. [137]
Ginsenoside metabolite	NCI-H716 cells	Elevated secretion of GLP-1; elevated plasma GLP-1; elevated tolerance to glucose	Kim et al. [129]
Myricetin	streptozotocin-administered rats; high-fat diet rats	Elevated plasma GLP-1; reduced tissue and plasma DPP-4	Lalitha et al. [138]
Luteolin	High-fat diet mice	Elevated GLP-1 in plasma, elevated tolerance to glucose	Kwon and Choi [139]

**Table 4:** Some GLP-1R agonist-induced β-cell survival proteins.

Name of Protein	Role of Protein	Reference
Protein kinase B	AKT, a serine/threonine kinase, exerts its effects by activating CREB, PDX1, and the mammalian target of rapamycin (mTOR) complex 1. Additionally, it inhibits glycogen synthase kinase 3 (GSK3β), caspase-9, FoxO1, and the Bcl-2-associated death promoter (Bad)	Camaya et al. [145]
ERK1/2	The Ras-dependent extracellular signal-regulated kinase 1 (ERK1)/2 mitogen-activated protein (MAP) kinase pathway plays a role in regulating cell survival.	Quoyer et al. [146]
MAPK10/JNK3	Anti-apoptotic mechanisms involving unidentified targets are present. JNK3 is regulated by MAP8IP1/JIP-1/IB1	Ezanno et al. [144]
SERCA2b	A P-type ATPase that regulates endoplasmic reticulum (ER) Ca2+ stores is responsible for maintaining calcium ion levels within the ER	Lee [147]; Tong et al. [148]
CREB	A transcription factor that enhances the expression of insulin receptor substrate 2 (IRS2), which is essential for IGF-1 and insulin receptor signaling, ultimately resulting in AKT activation.	Jhala et al. [149]
MAK8IP1 also called Islet Brain 1/JIP1	MAPK8IP1 functions as a scaffold protein that anchors MAP3K/MAP2K/JNK in the anti-apoptotic JNK signaling pathway. This scaffold protein plays a crucial role in coordinating and facilitating the signaling cascade to prevent apoptosis.	Tenenbaum et al. [150]

## Data Availability

The data presented in this study are available upon request from the corresponding author.
